# Evaluation of a novel handheld point-of-care ultrasound device in an African emergency department

**DOI:** 10.1186/s13089-020-00200-8

**Published:** 2020-12-07

**Authors:** Samuel L. Burleson, Jonathan F. Swanson, Erin F. Shufflebarger, Douglas W. Wallace, Matthew A. Heimann, James C. Crosby, David C. Pigott, John P. Gullett, Maxwell A. Thompson, Christopher J. Greene

**Affiliations:** Department of Emergency Medicine, University of Alabama at Birmingham, Old Hillman Building Suite 251, 619 19th St S, Birmingham, AL 35249 USA

**Keywords:** Point-of-care ultrasound, Handheld, Butterfly iQ, Resource-limited settings, Emergency, Tropical infectious disease

## Abstract

**Background:**

Many point-of-care ultrasound devices are now “pocket-sized” or handheld, allowing easy transport during travel and facilitating use in crowded spaces or in austere low-resource settings. Concerns remain about their durability, image quality, and clinical utility in those environments.

**Method:**

Five emergency physicians with training in point-of-care ultrasound employed the Butterfly iQ, a novel handheld ultrasound device, in routine clinical care in a busy, high-acuity African emergency department over a period of 10 weeks. We retrospectively evaluated the performance of the Butterfly iQ from the perspectives of both the clinicians using the device and expert ultrasound faculty reviewing the images.

**Results:**

We found advantages of the Butterfly iQ in a high-acuity African emergency department include its use of a single probe for multiple functions, small size, ease of transport, relatively low cost, and good image quality in most functions. Disadvantages include large probe footprint, lower, though still adequate, cardiac imaging quality, frequent overheating, and reliance on internet-based cloud storage, but these were surmountable. We also report a wide variety of patient presentations, pathology, and procedures to which the device was used.

**Conclusion:**

We conclude the Butterfly iQ is an effective, though imperfect, point-of-care ultrasound device in a low-resource emergency setting. We will continue to employ the device in clinical emergency care and teaching in this setting.

## Introduction

Newly-marketed point-of-care ultrasound (POCUS) devices like the Butterfly iQ (iQ, Butterfly Network, Inc, Guilford, CT, USA) have generated significant excitement over their potential in emergency departments (EDs), critical care units, and resource-limited settings (RLS). Potential benefits of the iQ include small size, lower cost, integration with the user’s mobile phone or tablet, and use of silicon-chip based technology obviating the need for multiple transducers. There has been little critical evaluation of the device itself, particularly in RLS. We describe our use of the Butterfly iQ in routine clinical operations in a busy, high-acuity African ED and review its performance and applicability to RLS.

## Personnel and setting

Five emergency physicians from the United States worked alongside the ED staff of a busy referral hospital in rural east Africa treating medical and surgical patients of all ages over a period of 10 consecutive weeks in the fall of 2019. All physicians have surpassed accepted POCUS training guidelines [1]. Two (SLB and JFS) have completed or are enrolled in a Point-of-care Ultrasound in Resource Limited Settings fellowship [2]. Advanced imaging is available, but access is limited by the need for payment prior to testing; in practice, patients often wait hours or days prior to imaging. Patients were selected by clinicians at the bedside if POCUS was indicated as a part of routine clinical care; therefore, informed consent was not required. Patients were scanned with the Butterfly iQ connected to an Apple iPad (iPad 5th Generation, Apple, Cupertino, CA, USA). Images were later reviewed for quality assurance by ultrasound fellowship-trained faculty. Final diagnoses were determined from a combination of the medical record, discussions with inpatient teams, and expert image review.

## Results and discussion

The Butterfly iQ performed well and met clinician needs for a POCUS machine in this single RLS. Its advantages over cart-based machines are magnified, where financial resources, floor space, and reliable power may be scarce. Advantages and disadvantages of the Butterfly iQ are summarized in Table 1.

**Table 1:** Advantages and disadvantages of the butterfly iQ

*ADVANTAGES*
Single probe replaces multiple traditional transducers, capable of many scan types
Image quality excellent compared to other handhelds
Low cost
User-friendly app
*DISADVANTAGES*
Cardiac imaging lower quality than other modes
Relatively frequent overheating
Single probe relatively heavy with large footprint, occasionally compressing small structures

### Advantages

**Fig. 1.**
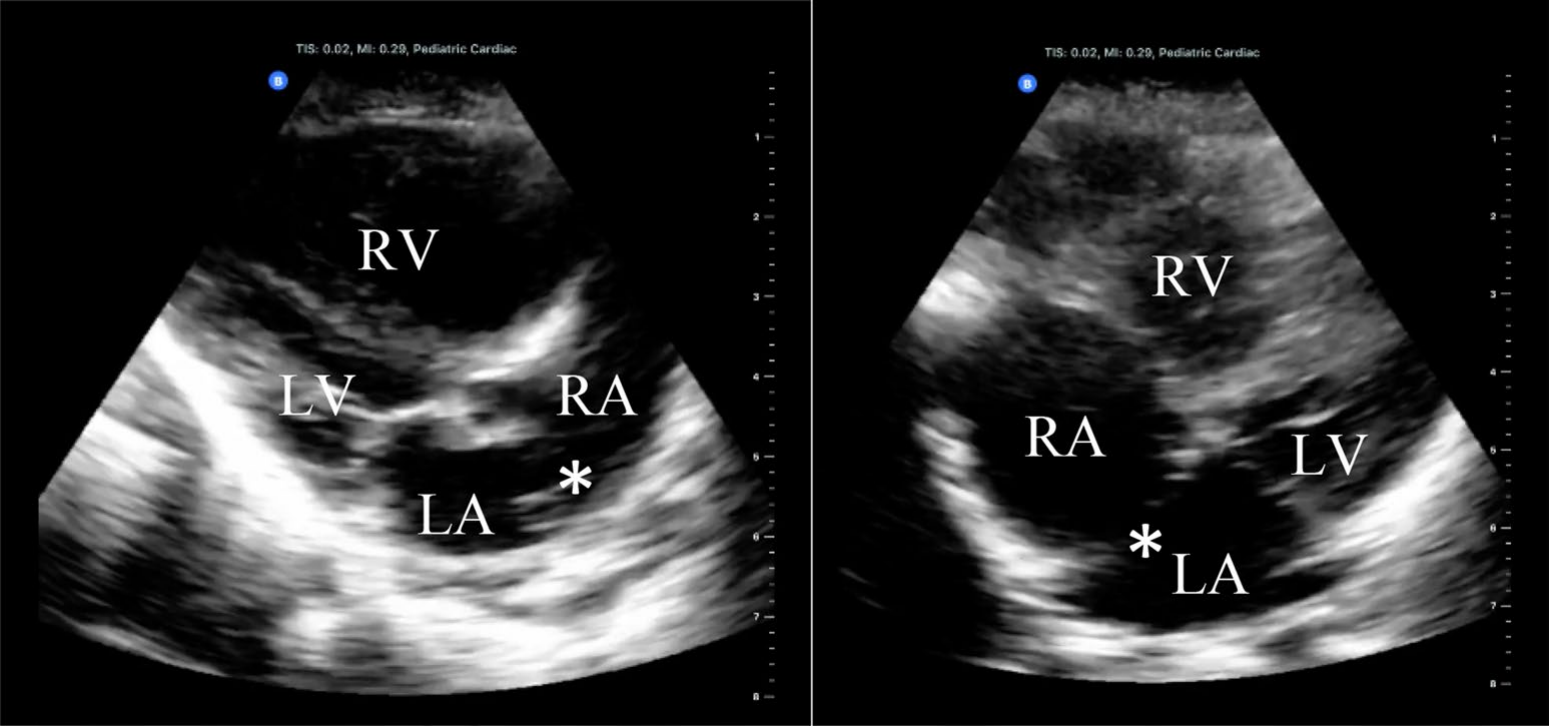
3-month-old male presenting with acute respiratory distress. POCUS revealed a dilated right atrium and ventricle with a prominent atrial septal defect, seen in parasternal long view (left) and a slightly modified apical four chamber view (right). LA, Left Atrium, LV, Left Ventricle, RA, Right Atrium, RV, Right Ventricle, *, Atrial Septal Defect

**Fig. 2:**
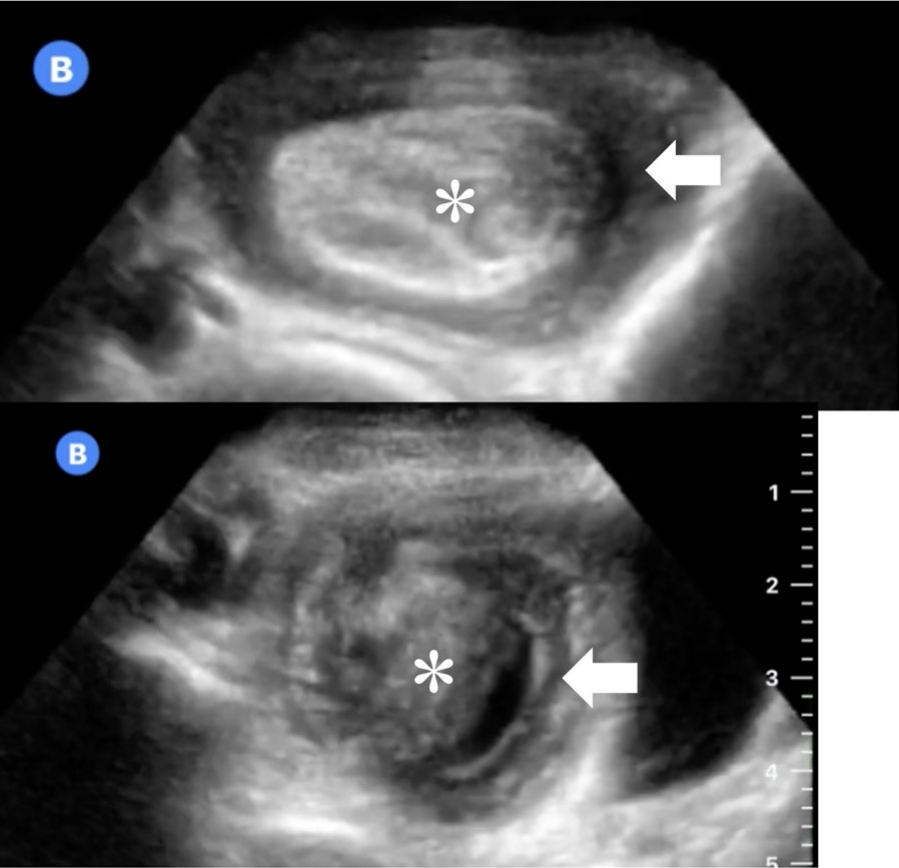
Abdominal point-of-care ultrasound of a previously healthy 3-year-old male with abdominal pain, nausea, and vomiting diagnosed with intussusception, confirmed and treated by air contrast enema. **a** reveals a stereotypical “target sign”, hyperechoic compressed inner loop of bowel (*) telescoping within a hypoechoic, edematous outer loop (arrow). **b** reveals the target sign in another cross sectional plane, with multiple layers of telescoping bowel

**Fig. 3:**
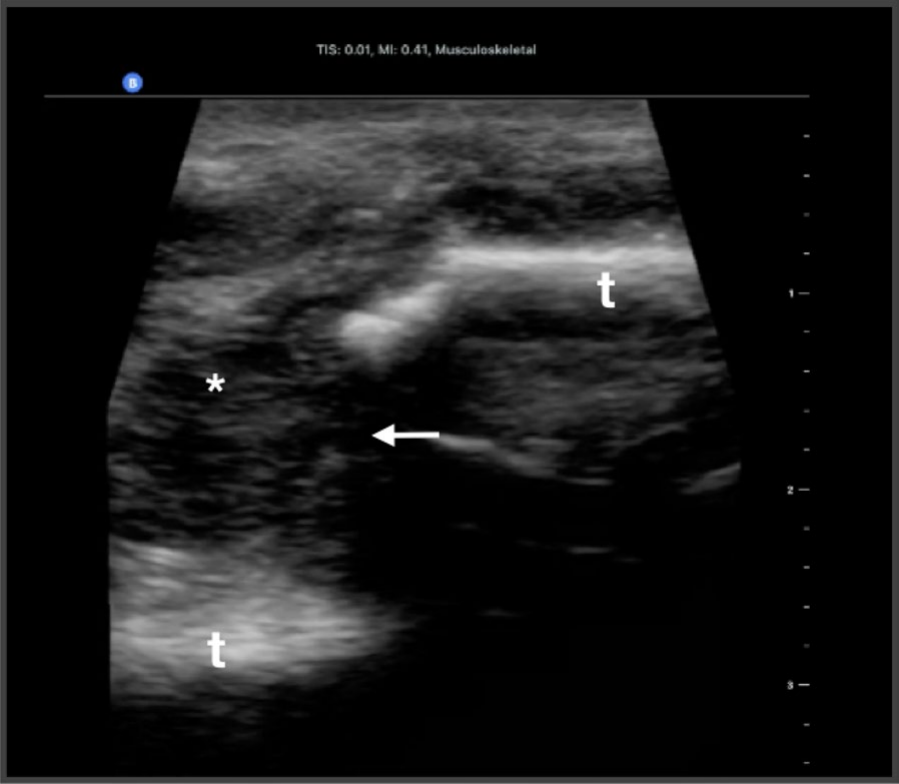
Musculoskeletal ultrasound of a young male patient with blunt trauma demonstrating two separate sections of tibial cortex (t), with displacement (arrows). Note the associated fracture hematoma visualized (*)

The combination of flexibility and mobility of a single probe with preset modes replacing multiple transducers is the paramount benefit of the device. We successfully employed the iQ in a wide variety of scans and patient presentations (see Table 2, Figs. 1, 2, 3) and procedural guidance (see Table 3).

**Table 2:** Point-of-care ultrasound findings using Butterfly iQ in African Emergency Department

Age/sex	Presentation	US exams	US findings
44 yo M	Afib RVR, Cardiogenic shock	Cardiac	Calcified left atrial thrombus
3 yo M	Abdominal pain, Fussy, Bloody stool	GI	Intussusception
28 yo F	Submandibular swelling	MSK	Submandibular abscess
25 yo M	Dyspnea on exertion, Hx of PCE	Cardiac, Lung	Large PCE without tamponade, Bilateral pleural effusions
Young Adult M	Leg pain	MSK	Mid-shaft femur fracture with displacement
23 yo F	Abdominal pain, Hx of abdominal mass	GI	Dermoid cyst (recurrent)
78 yo M	Early satiety, Rectal mass on exam	GI	Novel dx of diffuse hepatosplenic lesions concerning for malignancy
28 yo M	Necrotic finger	MSK	Abscess vs necrosis
3 mo M	Acute respiratory failure	Cardiac, Lung	Novel dx of Atrial Septal Defect
114 yo M	Dyspnea	Cardiac, Lung	Novel dx of HFrEF
13 yo F	Novel Afib and hypoxia, Hx of RHD	Cardiac	Massive MR, LA dilation, Small PCE (Consistent with known RHD)
14 yo M	Dyspnea, Anasarca on exam, Hx of RHD and malaria	Cardiac	HFrEF, TR with RA dilation, MR (Consistent with known RHD)
24 yo M	Left flank pain	GI	Splenic lesion (Subcapsular hematoma vs Infiltration)
1 yo M	Hypoxia, fever, sepsis	Lung	Bilateral B-lines
1 yo F	Hypoxia, Hx of Ventricular Septal Defect	Cardiac, Lung	RV Dilation and hypertrophy, Persistent VSD, Bilateral B-lines
74 yo F	Respiratory arrest	Cardiac	Dilated/poorly contractile RV, Full IVC
2 mo F	Failure to thrive	CNS	Hydrocephalus
Young Adult M	Leg pain	MSK	Mid-shaft tibia fracture with displacement
Elderly Adult M	Recent DVT, Dyspnea	Cardiac	Right heart strain
20 yo F	2-week post-partum, Peritonitis on exam	GI	Pelvic free fluid
28 yo M	"Hematemesis" found to be hemoptysis, Hypoxia	Cardiac, Lung, GI	Splenic lesion, Bilateral B-lines, Normal LV Ejection Fraction
Elderly Adult M	Dyspnea, Anasarca on exam, Hypoxia	Cardiac, Lung	Dilated RA and RV, TR, Bilateral B-lines, Pleural effusions, Ascites
23 yo F	Suicide attempt by drowing, Third trimester pregnancy	OB	Normal Fetal heart rate and Fetal movement
7 yo M	Abdominal pain, vomiting	GI	SBO from worm burden
25 yo M	Dyspnea and chest pain with near-syncope, Novel Afib with RVR	Cardiac	Thickened Mitral Valve, Dilated LA, Massive MR (Suspected RHD)
5 yo M	Abdominal distention	GI	Enlarged bladder with mild hydronephrosis
3 yo F	Constipation, Reports of “worms in stool”	GI	SBO, No parasites visualized
85 yo M	RUQ abdominal pain and jaundice	GI	Intrahepatic biliary dilation, RUQ Mass
35 yo F	Chest pain, Hx of Tuberculosis	Lung	Loculated pleural effusion
37 yo M	Abdominal pain, Constipation	GI	SBO, Dilated gallbladder
76 yo M	Hypoxia and sepsis	Lung	Subpleural consolidations and B-lines
19 yo F	Hx of tamponade on outpatient echocardiogram	Cardiac	Large PCE, No tamponade
25 yo M	Hypoxia, Novel Afib with RVR	Cardiac	Massive LA dilation and MR, Thickened anterior mitral valve leaflet (Suspected RHD)

Afib, Atrial fibrillation; CNS, Central Nervous System; Dx, Diagnosis; HFrEF, Heart Failure with Reduced Ejection Fraction; GI, Gastrointestinal/Genitourinary; Hx, History; IVC, Inferior Vena Cava; LA, Left Atrium; MR, Mitral Regurgitation; MSK, Musculoskeletal/Soft tissue; OB, Obstetric; PCE, Pericardial Effusion; RA, Right Atrium; RHD, Rheumatic Heart Disease; RUQ, Right Upper Quadrant; RV, Right Ventricle; RVR, Rapid Ventricular Response; SBO, Small Bowel Obstruction; TR, Tricuspid Regurgitation

**Table 3:** Procedures guided by Butterfly iQ in an African Emergency Department

“Easy IJ”—peripheral IV cannula placed in internal jugular vein for short-term resuscitation [4]
Peripheral IV cannulation
Endotracheal tube placement [5]
Foreign body localization and removal
Paracentesis
Thoracentesis

Image quality, particularly in the abdominal and musculoskeletal modalities, was excellent, though not generally up to the standards of a cart-based system. With few exceptions (see below), image quality in all modalities was adequate to answer the clinical question.

The software application is user-friendly, allowing rapid alternation between preset scanning modes, enabling multiple protocols on the same patient with only a few gestures. Some routine calculation functions (such as gestational age) were not available during our experience with the device. We found the increased screen size of an iPad compared to a mobile phone beneficial for most uses.

The cost of the device (approximately US$2000), plus a required $420 annual subscription fee to the cloud-based image storage) places it within reach of some individual clinicians, and many healthcare institutions, even in RLS. Battery life was adequate, usually enough to last an entire 12-h shift on a full charge, though we did not specifically measure continuous scanning time. When necessary, we were able to charge the device from main power on shift. Small, relatively inexpensive, third party solar-powered chargers could also provide additional charging.

### Disadvantages

Echocardiography habitually seemed lower quality than other modes. There was an apparent drop in resolution and frame rate, most noticeably associated with the use of color Doppler. Like other pocket-sized devices, the iQ also lacks spectral Doppler. Despite these limitations, we were able to detect significant cardiovascular pathology. All views were generally obtainable, adequate to guide resuscitation, and answer basic clinical questions pertinent to the RLS [3].

Images are stored on a cloud-based server requiring internet access to upload. Until they are uploaded, the images remain in an "outbox", where they can easily be deleted. Without reliable internet access, many images remained in this "outbox” for the duration of our field work, inhibiting our ability to share or review images.

We encountered several challenges related to the device’s hardware. The first was periodic overheating, rendering further scanning impossible until the device cooled. Overheating was not appreciably tied to any specific scanning mode or function. We mitigated this by briefly running water over the waterproof end of the device until cooled. We noticed a small rubber seal loosening near the end of the transducer by the closing of our field experience, without any discernible change in function. We found the cord length (125 cm) slightly short, especially when performing POCUS-guided procedures. The probe itself weighs 0.7 lb (0.3 kg), more than twice most other transducers, and its footprint is larger than a phased array probe, which was occasionally problematic when placing ultrasound-guided peripheral IVs and scanning between ribs, respectively. This may have contributed to the decreased quality of some echocardiographic images.

## Limitations

These findings represent a retrospective review of the authors’ personal experiences with the Butterfly iQ device during routine clinical work in an African ED in an attempt to evaluate its performance in RLS. Patients were scanned at the discretion of the clinician at the bedside or because of restricted access to other diagnostics, introducing the possibility of selection bias. No patient-oriented outcomes were assessed and no comparisons between devices were available.

## Conclusion

The Butterfly iQ was employed in a wide variety of patient presentations, scanning indications, and procedural guidance in a busy, high acuity RLS ED. Advantages include its use of a single probe for multiple functions, small size, relatively low cost, and good image quality in most functions. Disadvantages include large probe footprint, lower, though adequate, cardiac imaging quality, frequent overheating, and reliance on internet-based cloud storage, but these were surmountable. We believe the iQ is an effective POCUS device for emergency care in the RLS and we will continue to employ it for patient care and clinical teaching.

## Data Availability

All ultrasonographic data is stored on a proprietary cloud-based storage system, as detailed in the text.

## Abbreviations

ED: Emergency department
POCUS: Point-of-care ultrasound
RLS: Resource-limited settings
